# Supplementary material for: Bioprospection of the Antarctic Diatoms Craspedostauros ineffabilis IMA082A and Craspedostauros zucchelli IMA088A
Source: Mar Drugs. 2024 Jan 6;22(1):35. doi: 10.3390/md22010035 (PMC10820014; doi:10.3390/md22010035)
Supplement: Supplementary file 1 [file marinedrugs-22-00035-s001.zip › Table S1.pdf]

Table S1. Antioxidant activities of the acetone and methanol extracts of *C. ineffabilis* IMA082A and *C. zucchelli* IMA088A. The results are expressed as antioxidant activity (% of activity) at the concentration of 10 mg/mL. For the same column, different letters indicate significant differences (Multiple Comparisons of Means: Tukey Contrast, 95% family-wise confidence level). Values represent the mean ± standard error of mean (SEM) performed six times (n = 6); * positive control tested at 1 mg/ml. [1] BHT: Butylated hydroxytoluene; [2] EDTA: ethylenediamine tetraacetic acid.

Species	Extract	DPPH	ABTS	FRAP	CCA	ICA
C. ineffabilis IMA082A	Acetone 80%	14,55±0,62d	67,28±4,41b	26,33±4,49b	46,33±3,51b	85,05±1,87c
	Methanol 50%	6,96±1,11b	42,58±5,71a	22,90±3,03b	24,86±1,89a	89,73±9,36c
C. zucchelli IMA088A	Acetone 80%	11,02±1,85c	36,24±4,65a	26,02±4,41b	26,84±1,43a	5,91±2,59a
	Methanol 50%	4,79±0,94a	39,49±5,29a	13,37±1,49a	29,83±7,42a	26,81±4,46b
BHT *[1]		83,55±0,79e	94.44±0,20c			
EDTA *[2]					95,23±0,41c	96.08±0,45cd
