# Supplementary material for: Bioprospection of the Antarctic Diatoms Craspedostauros ineffabilis IMA082A and Craspedostauros zucchelli IMA088A
Source: Mar Drugs. 2024 Jan 6;22(1):35. doi: 10.3390/md22010035 (PMC10820014; doi:10.3390/md22010035)
Supplement: Supplementary file 1 [file marinedrugs-22-00035-s001.zip › Table S2.pdf]

Table S2. Enzymatic inhibitory properties of the acetone and methanol extracts *C. ineffabilis* IMA082A and *C. zucchelli* IMA088A. Results are expressed as inhibitory activity (% of inhibition) at the concentration of 10 mg/mL. For the same column, different letters indicate significant differences (Multiple Comparisons of Means: Tukey Contrast, 95% family-wise confidence level). Values represent the mean ± standard error of mean (SEM) performed six times (n = 6); * positive control tested at 1 mg/ml; ** positive control tested at 10 mg/mL.

Species	Extract	AChE	BChE	α-glucosidase	α-amylase	Lipase	Tyrosinase
C. ineffabilis IMA082A	**Acetone 80%**	61,05±3,58b	82,96±7,73c	19,20±1,20c	68,24±2,67c	18,28±3,25b	1,08±0,67a
	**Methanol 50%**	61,57±4,25b	42,20±5,16a	1,09±0,65a	1,07±1,16a	10,90±2,85a	13,69±4,70b
C. zucchelli IMA088A	**Acetone 80%**	46,21±3,66a	55,93±7,24b	16,74±0,49b	38,25±4,47b	41,86±4,58c	0,93±0,36a
	**Methanol 50%**	60,59±4,05b	65,12±1,09b	2,61±1,39a	1,89±2,61a	11,56±2,27ab	15,75±1,17b
**Galanthamine ***		91,31±0.38c	78.85±2.72c				
**Acarbose ****				73.45±1.00d	69.21±3.43c		
**Orlistat ***						62.65±3.14d	
**Arbutin ***							91.36±0.79c
