# Supplementary material for: Bioprospection of the Antarctic Diatoms Craspedostauros ineffabilis IMA082A and Craspedostauros zucchelli IMA088A
Source: Mar Drugs. 2024 Jan 6;22(1):35. doi: 10.3390/md22010035 (PMC10820014; doi:10.3390/md22010035)
Supplement: Supplementary file 1 [file marinedrugs-22-00035-s001.zip › Table S3.pdf]

Table S6. Fatty acid profiles of *C. ineffabilis* IMA082A and *C. zucchelli* IMA088A. The results are expressed as fatty acid methyl ester percentage. Values represent the mean ± standard error of mean (SEM) performed three times (n = 3); nd: not detected. [1]∑ SFA: Saturated fatty acids; [2]∑ MUFA: Monounsaturated fatty acids; [3]∑ PUFA: Polyunsaturated fatty acids. Asterisks indicate statistically significant differences ('***' = p–value < 0.001; '**' = p–value < 0.01 and '*' = p–value < 0.05).

FAME	*C. ineffabilis* IMA082A	*C. zucchelli* IMA088A	p–value
**C14:0**	10,12±0,19	8,88±0,22	0.002 **
**C16:1**	33,60±0,26	24,97±1,40	< 0.001 ***
**C16:0**	19,31±0,28	23,64±0,45	< 0.001 ***
**C18:3**	5,13±0,35	7,71±0,11	< 0.001 ***
**C18:2**	3,36±0,41	4,58±0,39	0.020 *
**C18:1**	0,95±0,026	0,85±0,47	0.725
**C18:0**	1,72±0,05	3,60±0,11	< 0.001 ***
**C20:4n-6**	22,30±0,39	16,47±0,29	< 0.001 ***
**C22:6n-3**	1,49±0,14	1,85±0,25	0.095
**C22:0**	nd	0,80±0,15	–
**C24:1**	1,77±0,10	5,04±0,39	< 0.001 ***
**C24:0**	0,28±0,01	1,62±0,23	< 0.001 ***
**∑ SFA [1]**	31,42±0,11	38,54±0,58	< 0.001 ***
**∑ MUFA [2]**	36,32±0,37	30,86±0,85	< 0.001 ***
**∑ PUFA [3]**	32,28±0,29	30,60±0,55	0.009 **
